# Safety of Redo Hepatectomy for Colorectal Liver Metastases after Selective Interarterial Radiation Therapy: A Case Report

**DOI:** 10.1155/2014/712572

**Published:** 2014-03-04

**Authors:** Kyriakos Neofytou, Harpreet Wasan, Satvinder Mudan

**Affiliations:** ^1^Royal Marsden Hospital, Department of Academic Surgery, Upper GI/HPB Unit, Fulham Road, London SW3 6JJ, UK; ^2^Oncology, Imperial College Healthcare NHS Trust, Hammersmith Hospital, Du Cane Road, London W12 0HS, UK

## Abstract

Surgical resection is the only potentially curative strategy in the treatment of patients with colorectal liver metastases (CLM). Unfortunately, only about 10%–15% of patients are candidates for resection. Preoperative chemotherapy aims to increase the number of patients that may be eligible for liver resection by downsizing liver metastases. For patients with unresectable, chemotherapy refractory CLM the available treatment options are limited. Selective interarterial radiation therapy (SIRT) is one of the most promising treatment options for this group of patients. Although only a small number of these patients have been reported as becoming candidates for potentially curative hepatic resection following sufficient reduction in the volume of liver metastases, the question arises regarding the safety of liver resection in these patients. We report a case of a patient who presented unresectable liver relapse of CLM after previous right hepatectomy. He underwent SIRT which resulted in downsizing of the liver metastases making the patient candidate for left lateral sectionectomy. He underwent the redo hepatectomy without any complications. To the best of our knowledge, this is the first reported case of redo hepatectomy after SIRT for CLM.

## 1. Introduction

Colorectal cancer (CRC) is the third leading cause of cancer-related death [1]. The liver is the most common site of metastatic spread in colorectal cancer (CRC). Approximately half of patients experience liver metastases during the course of their disease [2, 3]. Liver metastases from colorectal cancer are the main cause of morbidity and mortality among this patient group [4]. Liver resection has been established as the treatment of choice for these patients, and with the appropriate selection of patients 5-year survival rates approach 35% to 40% [5, 6]. Instead, the median survival for nonsurgically treated colorectal metastases ranges from 5.7 to 19 months and for patients receiving no treatment average survival is just 7.4 months [7, 8].

Despite surgical advances, only 10% to 15% of patients have resectable liver disease at presentation [9, 10]. Preoperative chemotherapy has been introduced to increase the number of patients that may be eligible for liver resection by downsizing liver metastases [11]. Unfortunately, a large proportion of patients with unresectable CLM will experience disease progression during the course of neoadjuvant chemotherapy. For these patients, the available treatment options are limited.

Radioembolization (RE) or Selective interarterial radiation therapy (SIRT) is emerging as an important and useful locoregional treatment option in patients with unresectable, chemotherapy refractory CLM [12, 13]. It has also been used simultaneously with chemotherapy. In such a case the use of RE aims to enhance treatment-related response and prolong interval to disease progression compared with chemotherapy alone [14, 15]. A recent meta-analysis of this treatment modality as a treatment option for patients with CLM who had progressed disease despite treatment with “first line” chemotherapy showed promising results, with a high response rate of approximately 80% of these patients [16].

Potentially curative hepatic resection following sufficient reduction in the volume of liver metastases by RE has been described but has only been possible in a minority of colorectal metastases cases. So far, fewer than ten cases of liver resection after RE for CLM have been reported [17–21]. Consequently, our knowledge of how the RE affects the postoperative complications and especially the function of future liver remnant is very limited. On the other hand, as an increasing number of patients undergo RE more and more patients will present sufficient reduction in the volume of their liver metastases and will be candidates for potentially curative hepatic resection.

We report the first case of redo hepatectomy after RE for CLM.

## 2. Case Report

**Figure 1:**
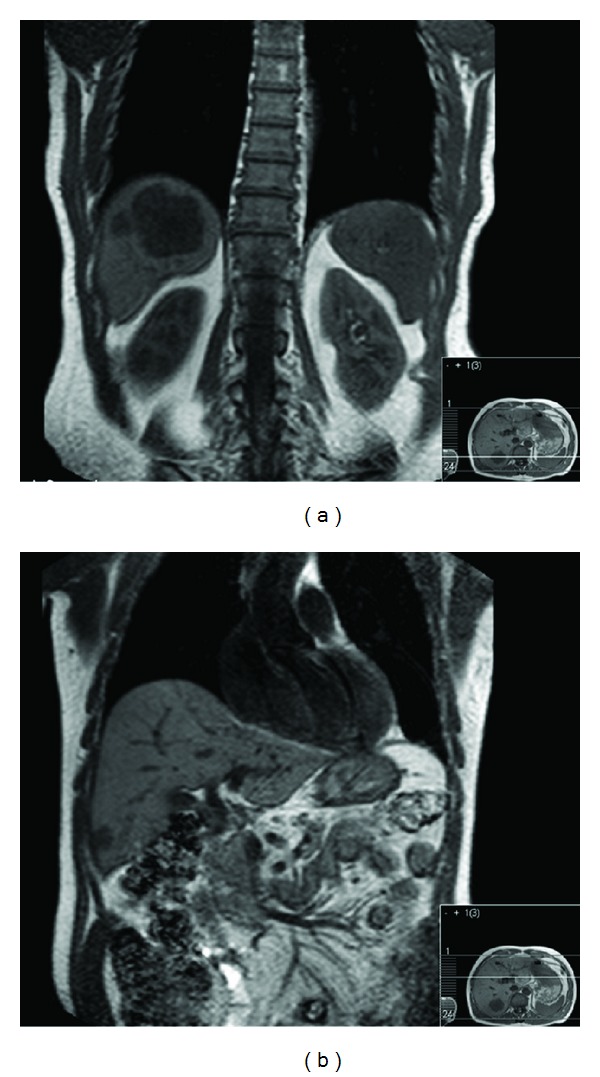
Synchronous liver metastases within right lobe of the liver. (a) Two lesions in segment VII measuring 43 mm and 14 mm in diameter. (b) A lesion close to the surface of segment V measuring 16 mm in diameter.

Our patient, a 63-year-old man, was initially diagnosed with an adenocarcinoma of the sigmoid colon 4 years ago. His staging CT scan showed the presence of unilobar synchronous liver metastases but no evidence of extrahepatic disease. The subsequent MRI scan showed 3 metastases within the right lobe of the liver. Two lesions were in segment VII measuring 43 mm and 14 mm in diameter. A further lesion measuring 16 mm in diameter was demonstrated close to the surface of segment V (Figure 1).

**Figure 2:**
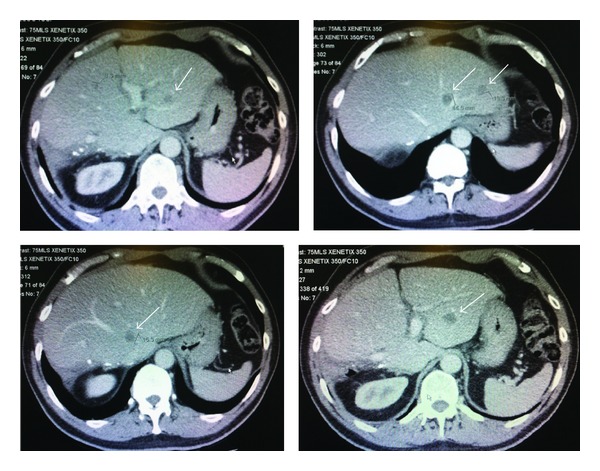
Recurrent unresectable liver metastases following right hepatectomy. Five new liver metastases (white arrows) in the remnant liver (Seg II, III, and IV).

The patient underwent laparoscopic sigmoid colectomy because of tumor bleeding. After the operation, he was given 4 cycles of Oxaliplatin/Capecitabine chemotherapy. The restaging MRI scan at the end of this period demonstrated disease progression (the liver metastases demonstrated an interval increase in size with the bigger one measuring 56 mm, but no new lesions were demonstrated). Because of the disease progression, he received second line chemotherapy consisting of FOLFIRI (6 cycles) with stable disease at the end of this treatment according to response evaluation criteria in solid tumors (RECIST). He underwent a right hepatectomy and 6 further cycles of FOLFIRI. After the 6th postoperative cycle of chemotherapy with FOLFIRI, the patient had an MRI scan which showed recurrent disease in the liver, with 5 new metastases (Figure 2). Two of these were in close proximity to the left hepatic vein, and as a consequence the liver resection was excluded as a treatment option.

The MDT recommendation was for yttrium-90 radioembolization of the liver remnant. Pretreatment diagnostic angiography was normal without variants of arterial liver blood supply. During this procedure, the gastroduodenal artery and the right gastric artery were embolized to prevent ^90^Y-microspheres from being distributed to visceral organs other than the liver. Subsequently, a scintigraphy using macroaggregates of technetium-99m labeled human serum albumin (MAA) revealed a hepatic pulmonary shunt 6.6%. As the hepatic pulmonary shunt was <10%, the patient received the full dose of ^90^Y-microspheres which was calculated by the BSA Method (1.1 GBq). ^90^Y-microspheres were administrated to the whole liver remnant through the left hepatic artery. The patient tolerated the procedure fairly well, without major adverse side effects. Two months after RE, restaging shows normalization of CEA and good response in the liver with reduced size of the lesions within segments IVa and II.

**Figure 3:**
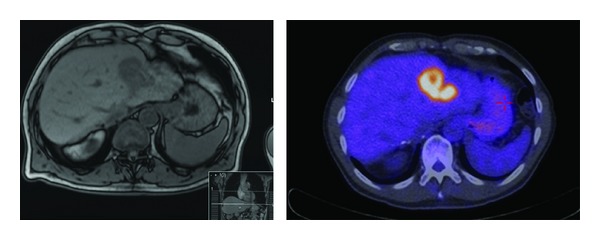
Solitary CRLM following selective interarterial radiation therapy (SIRT).

The patient received 6 cycles of low-dose irinotecan over the following 6 months. The PET scan at the end of this period was negative for liver metastatic disease. One year later, an MRI scan and a PET scan showed disease relapse within liver. The disease relapse consisted of a lesion measuring 5.7 cm in maximum dimension involving segments II and III. Segment IV was free of metastases and hypertrophic (Figure 3). The imaging results suggested that we should be able to achieve a resection of liver disease by a left lateral sectionectomy.

Despite our reservations regarding the possibly impaired liver regeneration and function after the hepatectomy, as a consequence of the previously multiple cycles of chemotherapy and especially as a consequence of the previously performed radioembolization of the whole remnant liver, we decided to proceed to the redo hepatectomy.

We proceeded with the left lateral sectionectomy. The postoperative course of the patient was unremarkable, and he was discharged on the 6th postoperative day. The postoperative values of bilirubin, albumin, INR, and LFTs were within normal limits.

## 3. Discussion

Radioembolization like all arterially directed liver therapies (intra-arterial hepatic chemotherapy [22], transarterial embolization [23], and transarterial chemoembolisation [24]) is based on an insight originating in the 1940's that in contrast to the normal liver parenchyma, which mainly relies on the portal vein, intrahepatic malignancies (primary and metastatic), takes its blood supply mainly from the arterial blood supply [25]. Radioembolization or SIRT (selective internal radiation therapy) is a form of arterially delivered brachytherapy aiming to target multiple sites of disease within the liver [26]. Radiation is delivered by radioactive isotopes labeled in microspheres that are injected into the arteries that feed the tumors through a transfemoral catheter. This catheter is advanced under fluoroscopic guidance into the hepatic artery branches that supply the metastatic tumours [27]. Radioembolization uses yttrium-90 (90Y), which is permanently bound to biocompatible, nonbiodegradable microspheres. These microspheres can be either glass or resin. Yttrium-90 is a pure-b emitter with a half-life of 2.67 days (64.2 h) which decays to stable zirconium-90. The microspheres preferentially lodge in the neovascular rim of the tumour(s) and deliver tumouricidal doses of radiation [28]. The minimal cellular inflammatory response within the tumors after the radioembolization indicates that the main mechanism of action of RE is the direct radiation injury of cancer cells which is a nonimmune mediated process [29].

It has been long established knowledge that the liver is very sensitive to external radiation therapy and patients may develop radiation-induced liver disease (RILD), months after an overdose of radiation [30, 31]. The symptoms of RILD comprise ascites, hepatomegaly, and elevated liver function tests [32]. Histopathologically, RILD is characterized by venoocclusive disease with congestion of the central veins and sinusoids [31–33].

In contrast to the external radiation which affects the whole or a big proportion of the liver, the mean tissue penetration of b-radiation which is emitted by yttrium-90 is 2.5 mm with a maximum range of 11 mm [28]. This fact, in combination with the observation that microspheres after RE are mainly identified in the vascular tumour bed, proposes that RE is much safer than external radiation regarding the radiation-induced normal liver parenchyma damage. However, the RE does not leave the normal liver parenchyma unaffected. Sangro et al. analyzed liver damage occurring after RE among 45 patients without previous chronic liver disease. 20% of these patients developed jaundice and ascites, which as mentioned above constitute characteristics of radiation-induced liver disease [34]. The liver biopsy of 2 of these patients showed hepatic venoocclusive disease. This new entity was named REILD (RE-induced liver disease) [34]. The harmful action of brachytherapy at normal liver parenchyma is also deduced from other publications which show that a significant percentage of patients who have undergone a RE show, even temporarily, disruption of liver function [20, 35].

The main risk factor for REILD is the prior to RE administration of chemotherapy, which in the case of patients with colorectal liver metastases who have undergone RE is a common component [34]. On the other hand, venoocclusive disease (VOD) is a fairly common adverse side effect of oxaliplatin-based combination chemotherapy one of the most frequently used regimens in patients with CLM [36].

Drawing on the above data together we can easily assume that patients who will undergo hepatectomy for CLM after multiple cycles of chemotherapy and RE are at increased risk of complications and especially at increased risk for developing liver failure if subjected to major hepatectomy. These data must always be taken into account before the decision of liver resection in patients with CLM who underwent RE. These data are even more important in the context of the above-described case as the decision to redo hepatectomy would equate with the decision that the future liver remnant would be only segments I and IV. Even apart from the RE, the danger of OVD was high enough as our patient had also received 18 cycles of oxaliplatin-based combination chemotherapy.

Our decision to proceed to redo hepatectomy was based mainly on (1) the excellent general condition of the patient (PS-0), (2) the presence of hypertrophic segment IV which provided sufficient volume of future liver remnant, (3) the absence of data arguing for significant damage of liver after RE (the procedure was well tolerated without adverse side effects and without evidence of impaired liver function (normal bilirubin, albumin, and INR levels)), and (4) the biological behavior of the disease which although initially was chemotherapy refractory, displayed good response to RE, and therefore the patient has had 18 months of benefit.

## 4. Conclusion

As more and more patients with unresectable, chemotherapy refractory CLM undergo RE, more and more patients will present sufficient reduction in the volume of their liver metastases and will be candidates for potentially curative hepatic resection. Although the existing data are not sufficient, they are enough to let us assume that many of these few patients will have OVD of their liver, putting them on a high risk for postoperative complications and liver failure (if they undergo major liver hepatectomy). The above reported case demonstrates that the proper selection of patients for hepatectomy after RE makes the operation safe even in cases of redo hepatectomies.
